# Supplementary material for: Acto-myosin force organization modulates centriole separation and PLK4 recruitment to ensure centriole fidelity
Source: Nat Commun. 2019 Jan 3;10:52. doi: 10.1038/s41467-018-07965-6 (PMC6318293; doi:10.1038/s41467-018-07965-6)
Supplement: Supplementary file 1 — Supplementary Information [file 41467_2018_7965_MOESM1_ESM.pdf]

Acto-myosin force organization modulates centriole separation and PLK4 recruitment to ensure centriole fidelity

Vitiello E. et al

Supplementary Materials:

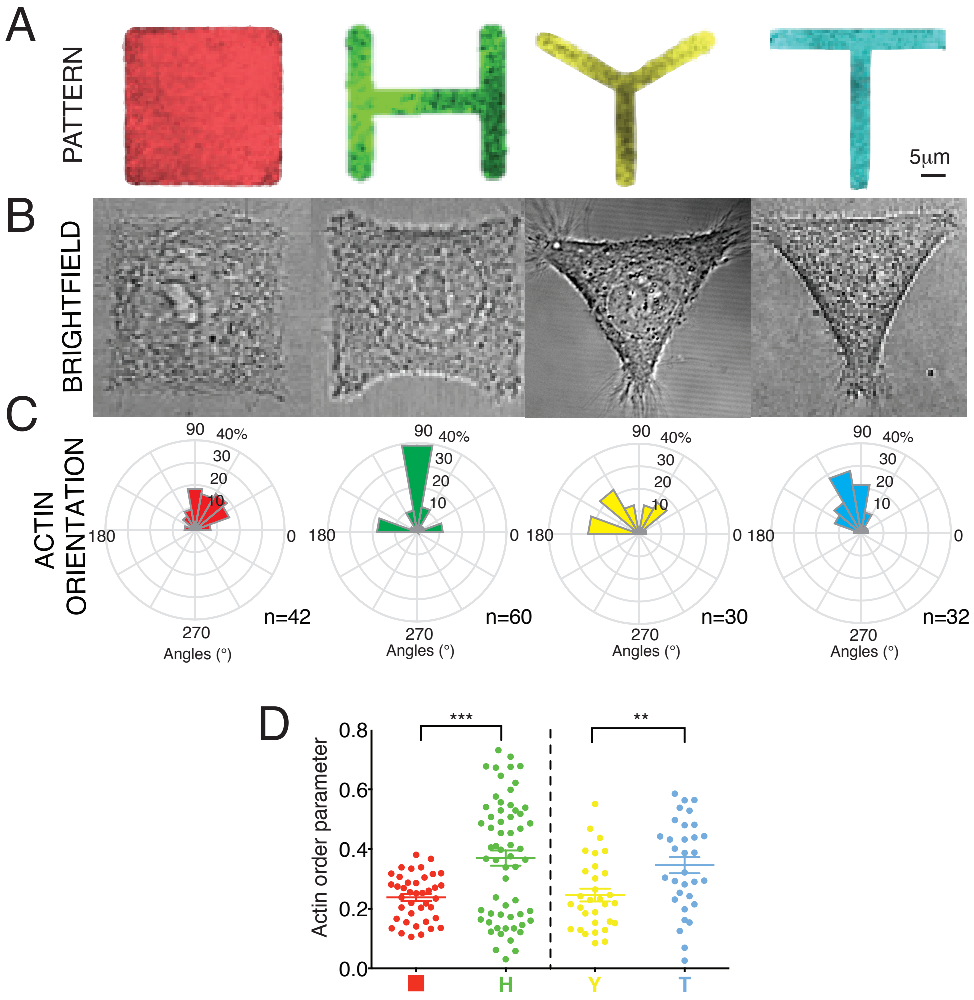

Supplementary Figure 1 Geometrical constraints define specific actin fiber orientation profiles.

(**A**) Fibronectin micropatterns. (**B**) Brightfield image of cells on micropatterns (**C**) Actin fiber main orientation determined for cells on Square (n=42), H (n=60), Tripod (n=30) and T micropatterns (n=32). Whereas Square and Tripod do not show a preferential orientation, H and T exhibit respectively 2 and 1 main actin fiber orientation axis. These angles are restricted to the [0°, 180°] range as actin fibers are not directed. (**D**) Actin order parameter plot. Actin order parameter quantifies the distribution of local orientations within a cell. As shown in the graph, cells plated on H and T shapes present more ordered actin architectures, compared to the less polarized shapes (Square and Tripod). Moreover, cells on H and T exhibit a higher content of long fibers that maintain their orientation throughout the cell. Error bars s.e.m. P-values were obtained with t-test; *** represents p<0.001 ** represents p<0.01 Scale bar: 5µm.

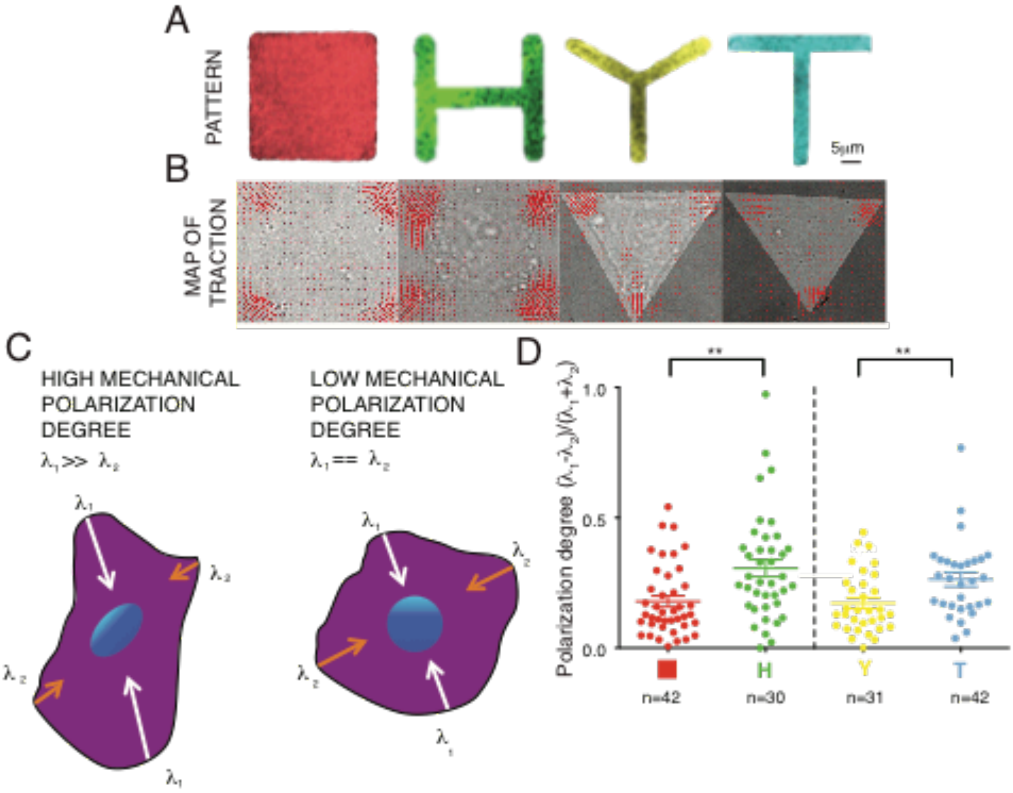

Supplementary Figure 2 Geometrical constraints induce precise mechanical polarization states.

(**A**) Fibronectin micropatterns. (**B**) Representative traction maps computed for cells adhering on 40KPa polyacrylamide hydrogels. (**C**) Schematic cartoons of cells with high and low mechanical polarization degree. λ_1 and λ_2 represent the two calculated eigenvalues of the first moment tensor. We defined mechanical polarization degree the ratio $(\lambda_1-\lambda_2)/(\lambda_1+\lambda_2)$. If it is close to zero, the force pattern is isotropic. If it approaches 1, the force pattern is highly oriented (uniaxial pinching). (**D**) Mechanical polarization degree plot. From the graph we can discriminate one group of shapes with lower mechanical polarization degree (Square and Tripod) and a second group with higher degree of polarization (H and T). Error bars represents s.e.m. P-values were obtained with t-test; ** corresponds to $p<0.01$. Scale bar: 5μm.

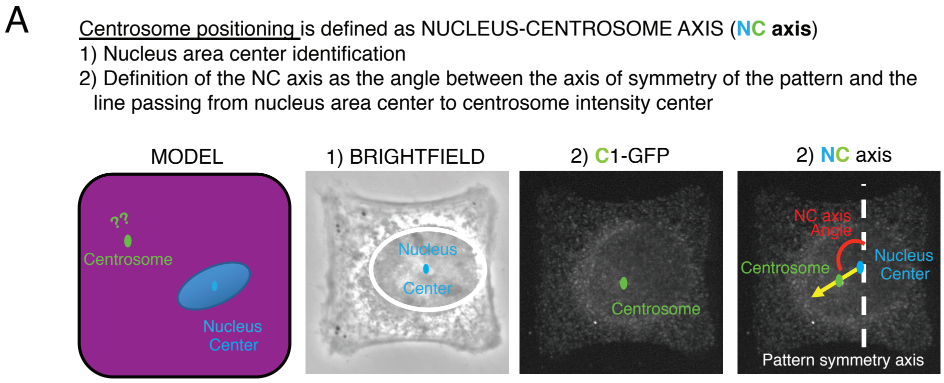

Supplementary Figure 3 Method adopted to quantify centrosome positioning

(**A**) Description of the method adopted to measure centrosome positioning. Centrosome positioning is defined as the orientation of the Nucleus-Centrosome axis (NC axis). The nucleus center is defined as the centroid of the nucleus region.

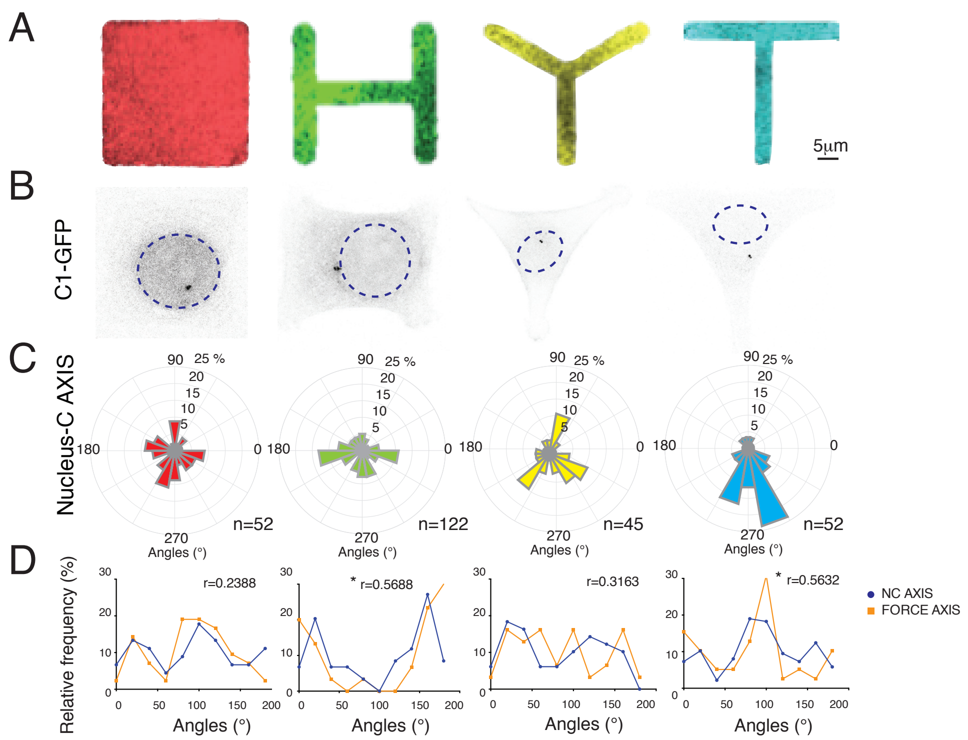

Supplementary Figure 4 Acto-myosin force axis controls centrosome-nucleus axis

(**A**) Fibronectin micropatterns. (**B**) Centrin1-GFP expression (C1-GFP). Dashed edge circle depicts the nucleus position within the presented cells. (**C**) Angle histogram of centrosome positioning, defined as the Nucleus-centrosome (Nucleus-C) axis orientation (see Supplementary Figure 3A for further explanation). Square (n=52) and Tripod (n=45) display random orientation, while 2 main axes for H (n=122) and 1 axis for T (n=52) are detected. These angles are restricted to the [0°, 360°] range as centrosome-nucleus axis is directed. (**D**) Correlation of angle distribution for Traction axis and centrosome-nucleus (NC) axis. Pearson test was used to estimate the correlation coefficient (r) and the statistical significance. P values for the r of H and T are 0.0450 and 0.0458 (Scale bar = 5µm).

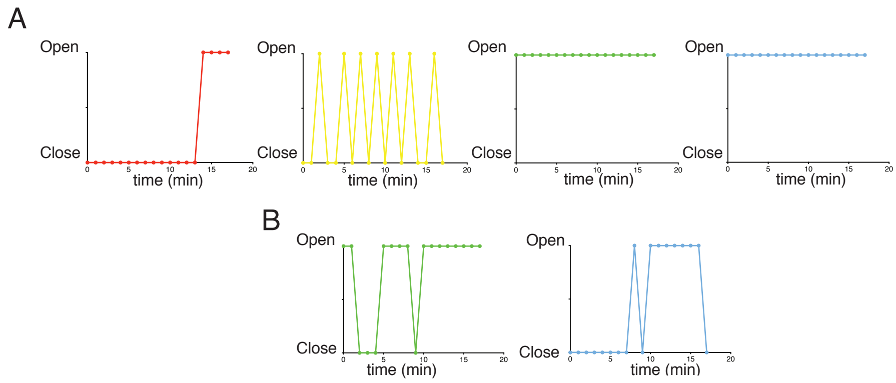

Supplementary Figure 5 Acto-myosin forces regulate centriole separation time

(**A**) Open-close time plot for the videos shown in Fig3A. (**B**) Open-close time plot for the videos shown in Fig4A,C.

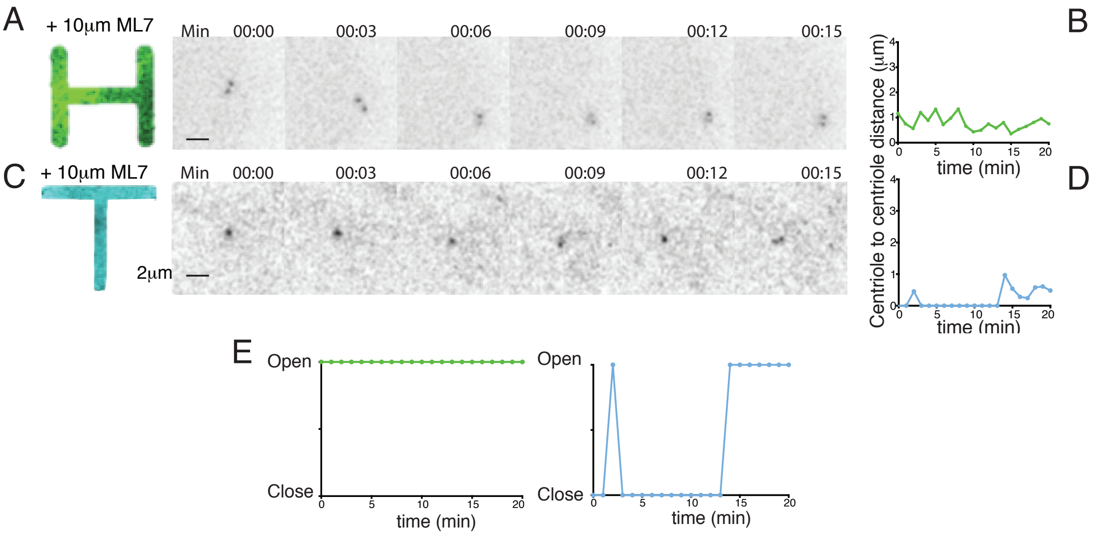

Supplementary Figure 6 Inhibition of actin contractility impairs centriole separation

Representative videos of C1-GFP HeLa cells plated on H (**A**) and T (**C**), treated with 10µM ML7 (**B**,**D**) Centriole-to-centriole distance plotted over time for the respective videos.. Time is in minutes (min). Images correspond to Movies 7-8. (**E**) Open-close plot for the videos shown in A,C. Scale bar: 2µm.

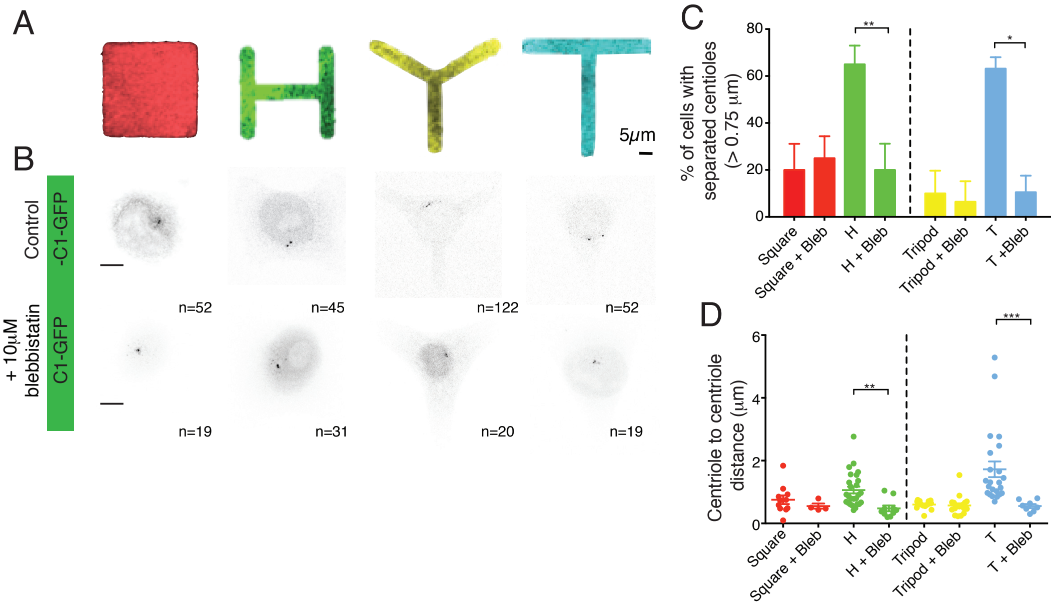

Supplementary Figure 7 Actin contractility inhibition with blebbistatin impairs centriole separation on shapes inducing high mechanical polarization

(**A**) Fibronectin micropatterns. (**B**) Representative images of control and blebbistatin-treated C1-GFP HeLa cells. Quantification of centriole separation frequency (**C**) and centriole-to-centriole distance (**D**) in Square (n=20), Square + Bleb (n=19), H (n=20), H + Bleb (n=20), Tripod (n=20), Tripod + Bleb (n=31), T (n=20), T + Bleb (n=19). Error bars represent SE in C and s.e.m. in D. Scale bar: 5µm. P-values were obtained with t-test; * represents $p<0.05$, ** $p<0.01$, *** $p<0.001$

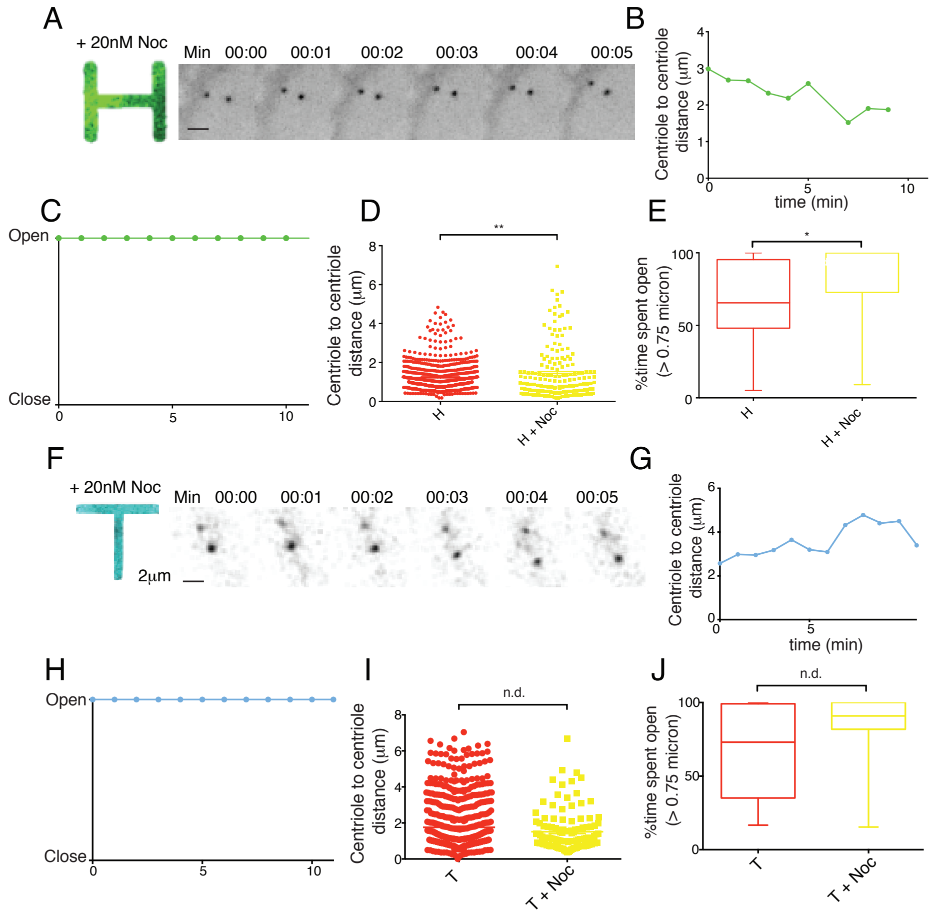

Supplementary Figure 8 Microtubule dynamics impairment does not significantly affect centriole separation

(**A**) Representative videos of C1-GFP HeLa cells plated on H, treated with 20nM Nocodazole (Noc). At this particular dose, microtubule dynamics is impaired without completely depolymerizing the network. Scale bar: 2µm. Images correspond to Supplementary Movie 9. (**B**) Centriole-to-centriole distance plotted over time for the video in A. (**C**) Open-close time plot for the videos shown in A. Centriole-to-centriole distance (**D**) and open time plot (**E**) for all the cells analysed (H n=46; H + Noc n=21). (**F**) Representative videos of C1-GFP HeLa cells plated on T, treated with 20nM Nocodazole (Noc). Scale bar: 2µm. Images correspond to Supplementary Movie 10. (**G**) Centriole-to-centriole distance plotted over time for the video in F. (**H**) Open-close time plot for the videos shown in F. Centriole-to-centriole distance (**I**) and open time plot (**J**) for all the cells analysed (T n=37; T + Noc n=13). P-values were obtained with t-test; * represents $p<0.05$, **. n.d. not different.

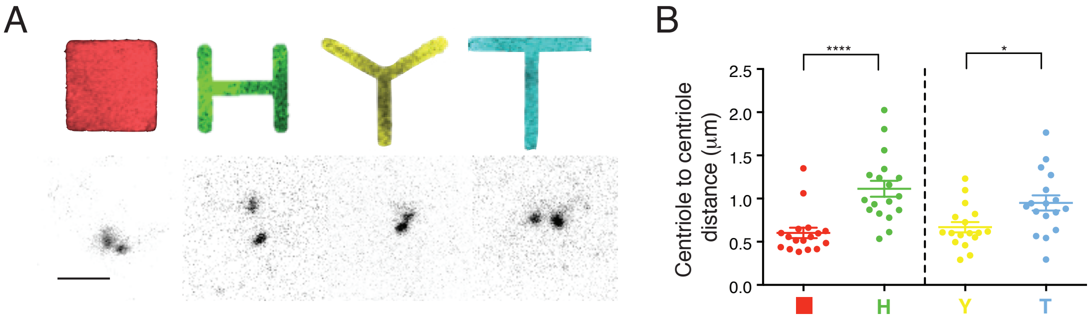

Supplementary Figure 9 Mechanical polarization controls centriole-to-centriole distance in disengaged centrioles during G_1

(**A**) Representative pictures of disengaged centrioles in G_1 synchronized cells on Square, H, Tripod and T. Scale bar: 2µm. (**B**) Centriole-to-centriole distance plot for Square (n=22), H (n=22), Tripod (n=22), and T (n=22) synchronized in G_1. As show in the graph, mechanical polarization control centriole-to-centriole distance specifically in G_1. P-values were obtained with t-test; **** represents $p<0.0001$ * corresponds to $p<0.05$.

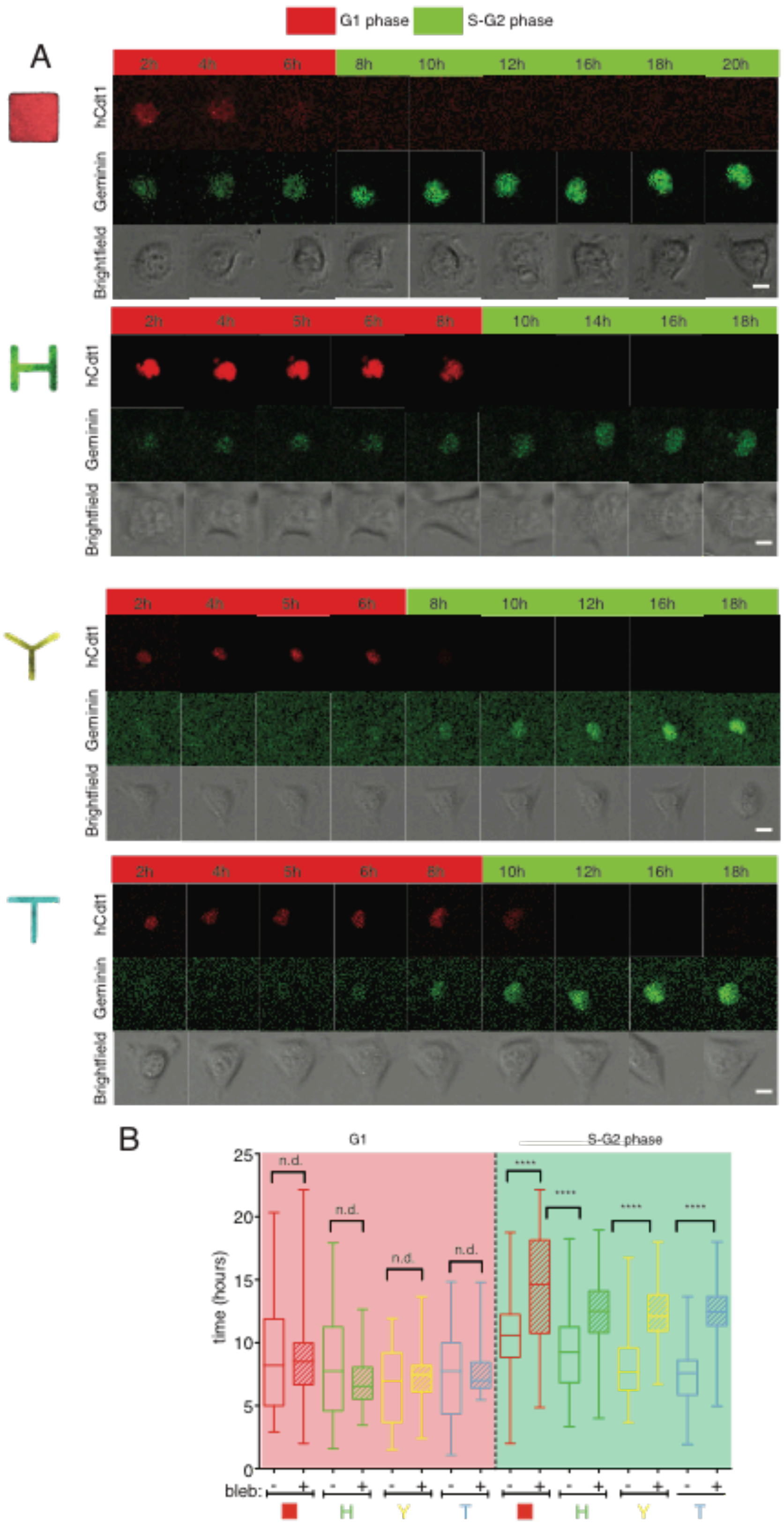

Supplementary Figure 10 Acto-myosin inhibition elongates S-G2 phase duration

(**A**) Representative hCdt1 (G_1-to-S phase reporter in red) and Geminin (S-to-G_2 phase marker in green) signal time-lapse for 10µm bleb-treated FUCCI HeLa cells plated on respective micropatterns. (Scale bar = 10µm). Time is in hours.

Images correspond to Supplementary Movies 15-18. (**B**) G_1/S and S-G_2 (to NEB: Nuclear envelope breakdown) time plot for all the FUCCI HeLa cells imaged over night after thymidine release. Whiskers represent minimum and maximum values. Box extends from the 25th to 75th percentiles. Line corresponds to median (Square n=90, Square+bleb=71, Tripod n=68, Tripod+bleb=106, H n=71, H+bleb=75, T n=53, T+bleb=90). Time is in hours. P-values were obtained with t-test; ** to $p<0.01$, **** to $p<0.0001$ From the graphs, we can observe how acto-myosin inhibition via blebbistatin treatment extend significantly S-G_2 phase length in all the shapes. n.d. not different.

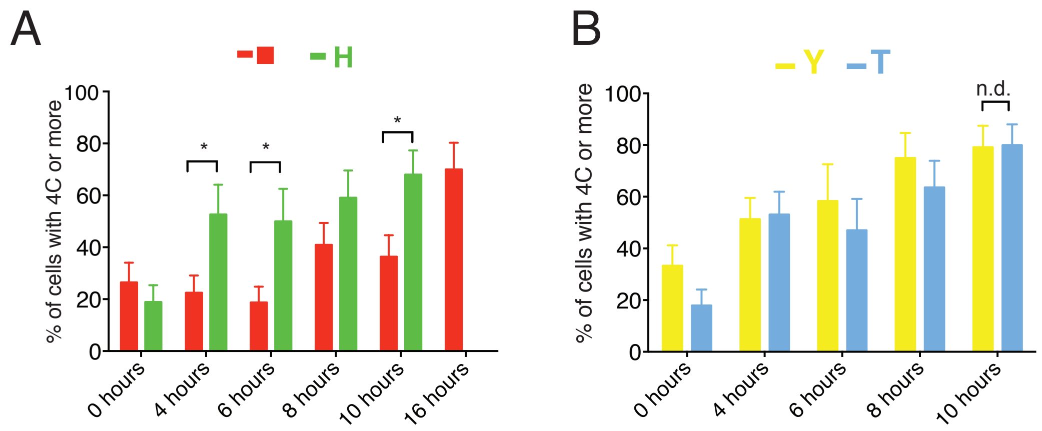

Supplementary Figure 11 Low mechanical polarization of Square delays centriole duplication

(**A,B**) Quantification of percentage of cells with 4 centrioles or more over time following thymidine release in C1-GFP cells plated on Square- H (A), Tripod-T (B). For each time-point 20-30 cells were analysed. As shown in the figures, whereas Tripod and T patterns do not display difference, Square shows a significant delay compared to H, with 70% of cells with 4 centrioles or more only after 18hours following release. Error bars represent s.e. P-values were obtained with t-test; * to $p<0.05$. n.d. not different.

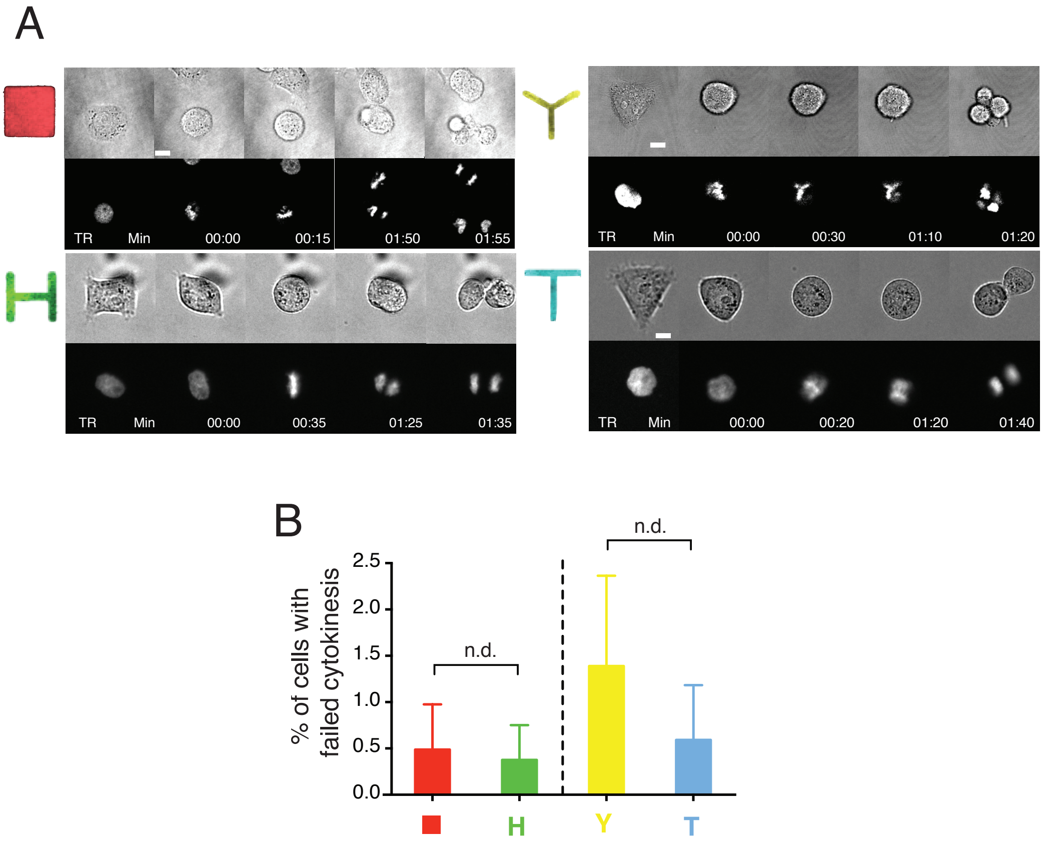

Supplementary Figure 12 Aberrant centriole duplication due to poor acto-myosin force organization is not caused by failed cytokinesis

(**A**) Representative frames from time-lapse movies of H2B-GFP HeLa cells analyzed in Fig. B. Scale bar = 10µm. TR represents the first time frame after thymidine release. Time zero corresponds to NEB. Images correspond to Supplementary Movies 19-22. (**B**) Percentage of cells with failed cytokinesis for Square (n=205), H (n=266), Tripod (n=144) and T (n=169). As shown in the picture, differences in mechanical polarization do not induce significant changes in the rate of cytokines failure. Error bars represent s.e.. P-values were obtained with t-test. n.d. not different.

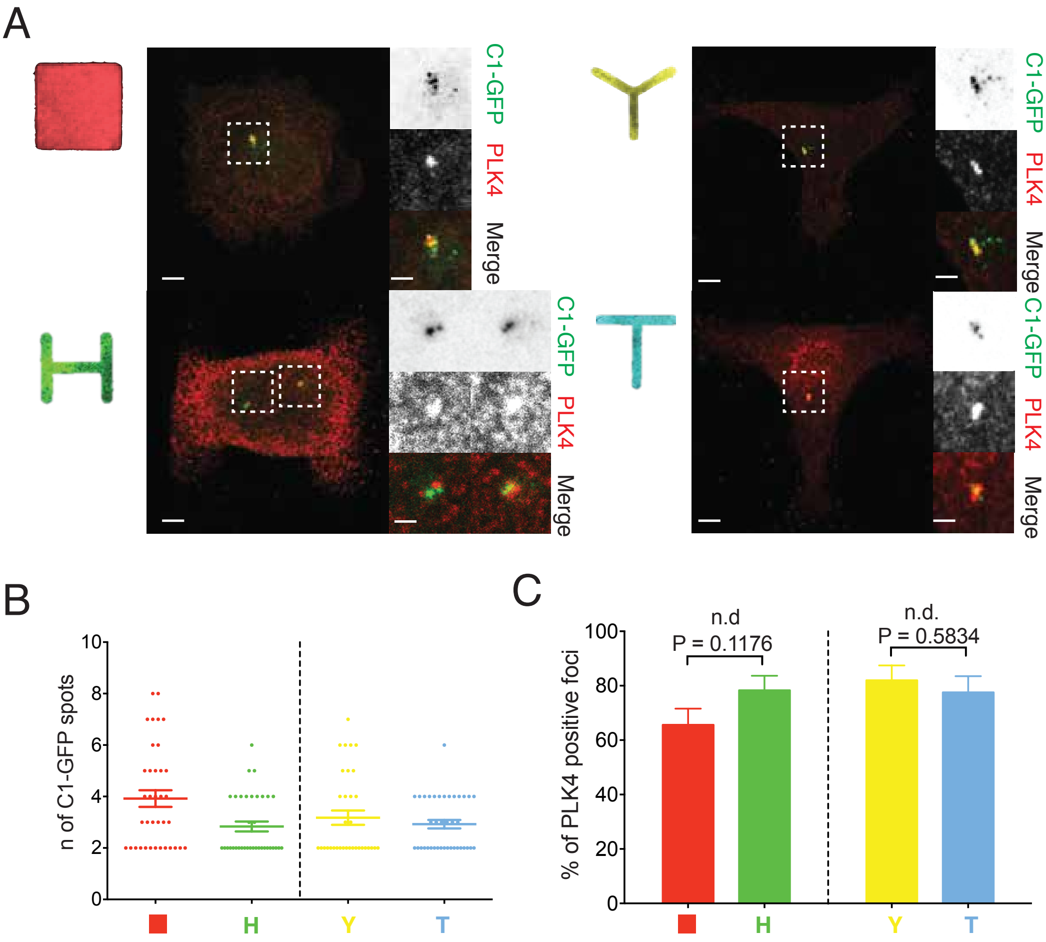

Supplementary Figure 13 Occurrence of centriolar satellites is low on all shapes

(**A**) PLK4 recruitment in C1-GFP HeLa cells plated on different micropatterns. Images show merge of fluorescence channels. Dashed boxes (scale bar = 5µm) indicate zoomed section presented in panels on the right of the pictures. Zoomed panels (scale bar = 2µm): from top to bottom, C1-GFP, total PLK4, Merge. (**B**) Chart representing the number of centrioles per cell on Square, H, Tripod and T micropatterns. As quantified in Fig 7B, cells on Square and Tripod present a higher percentage of cells with more than 4 centrioles when compared to cells on H and T. Square (n=37), H (n=36), Tripod (n=34) and T (n=39). (**C**) Percentage of PLK4 positive foci per cell. The plot show no differences among all the shapes, suggesting that even though cells on Square and Tripod have more than 4 centrioles, the majority of those are not centriolar satellites but aberrantly duplicated centrioles. Error bars represent s.e. P-values were obtained with t-test. n.d. not different.

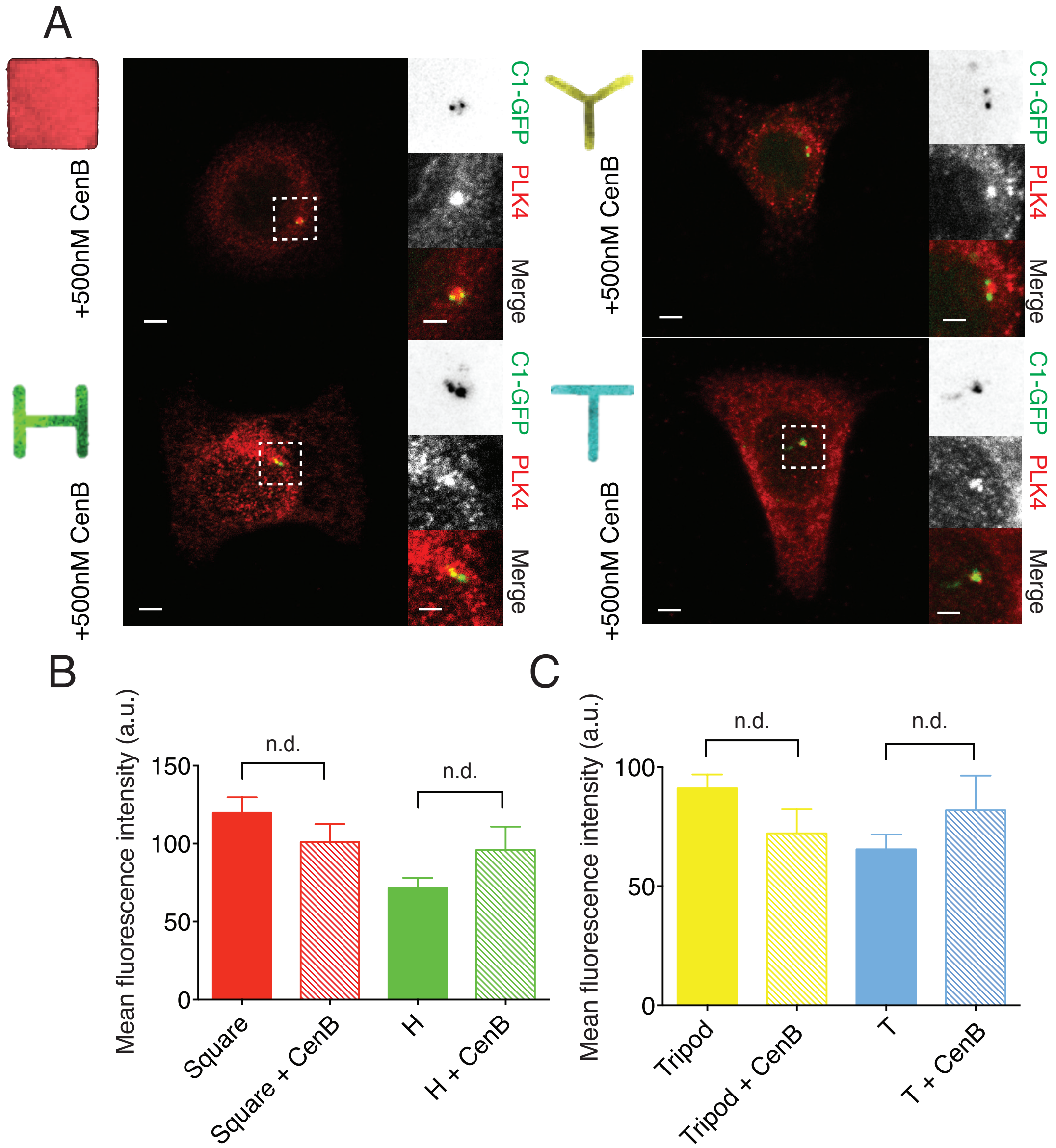

Supplementary Figure 14 CentrinoneB treatment does not affect PLK4 recruitment at the centrosome

(**A**) Representative images of PLK4 recruitment in C1-GFP HeLa cells plated on different micropatterns and treated with CentrinoneB (CenB), a PLK4 kinase inhibitor. Images show merge of fluorescence channels. Dashed boxes (scale bar = 5µm) indicate zoomed section presented in panels on the right of the pictures. Zoomed panels (scale bar = 2µm): from top to bottom, C1-GFP, total PLK4, Merge. (**B,C**) PLK4 mean fluorescent intensity for all untreated and CentrinoneB treated cells analyzed. Between 20 to 30 cells were analysed for all the conditions. Square and Tripod cells with lower mechanical polarization degree present higher PLK4 fluorescence intensity, indicating higher recruitment. No significant effect was induced by CentrinoneB (PLK4 kinase inhibitor), indicating that acto-myosin force-driven PLK4 localization does not depend on PLK4 kinase activity. P-values were obtained with t-test. n.d. not different.
