# Supplementary material for: Acto-myosin force organization modulates centriole separation and PLK4 recruitment to ensure centriole fidelity
Source: Nat Commun. 2019 Jan 3;10:52. doi: 10.1038/s41467-018-07965-6 (PMC6318293; doi:10.1038/s41467-018-07965-6)
Supplement: Supplementary file 3 — Description of Additional Supplementary Files [file 41467_2018_7965_MOESM3_ESM.pdf]

Description of Additional Supplementary Files

File Name: Supplementary Movie 1
Description: C1-GFP time-lapse in Square shape cell. Centrin1-GFP live imaging in HeLa cell on a square micropattern. Time is in hrs:min. Frame rate is 1min. Total acquisition length is 25min.

File Name: Supplementary Movie 2
Description: C1-GFP time-lapse in H shape cell. Centrin1-GFP live imaging in HeLa cell on a H micropattern. Time is in hrs:min. Frame rate is 1min. Total acquisition length is 30min.

File Name: Supplementary Movie 3
Description: C1-GFP time-lapse in Tripod shape cell. Centrin1-GFP live imaging in HeLa cell on a Tripod micropattern. Time is in hrs:min. Frame rate is 1min. Total acquisition length is 27min.

File Name: Supplementary Movie 4
Description: C1-GFP time-lapse in T shape cell Description: Centrin1-GFP live imaging in HeLa cell on a T micropattern. Time is in hrs:min. Frame rate is 1min. Total acquisition length is 27min.

File Name: Supplementary Movie 5
Description: C1-GFP time-lapse in H shape cell upon blebbistatin treatment Description: Centrin1-GFP live imaging in HeLa cell on a H micropattern upon inhibition of acto-myosin contractility via blebbistatin. Time is in hrs:min. Frame rate is 1min. Total acquisition length is 20min.

File Name: Supplementary Movie 6
Description: C1-GFP time-lapse in T shape cell upon blebbistatin treatment Description: Centrin1-GFP live imaging in HeLa cell on a T micropattern upon inhibition of acto-myosin contractility via blebbistatin. Time is in hrs:min. Frame rate is 1min. Total acquisition length is 29min.

File Name: Supplementary Movie 7
Description: C1-GFP time-lapse in H shape cell upon ML7 treatment Description: Centrin1-GFP live imaging in HeLa cell on a H micropattern upon inhibition of acto-myosin contractility via ML7. Time is in hrs:min. Frame rate is 1min. Total acquisition length is 30min.

File Name: Supplementary Movie 8
Description: C1-GFP time-lapse in T shape cell upon ML7 treatment. Centrin1-GFP live imaging in HeLa cell on a T micropattern upon inhibition of acto-myosin contractility via ML7. Time is in hrs:min. Frame rate is 1min. Total acquisition length is 40min.

File Name: Supplementary Movie 9
Description: C1-GFP time-lapse in H shape cell upon Nocodazole treatment. Centrin1-GFP live imaging in HeLa cell on a H micropattern upon inhibition of Microtubule dynamics via low dose Nocodazole treatment. Time is in hrs:min. Frame rate is 30sec. Total acquisition length is 10min.

File Name: Supplementary Movie 10

Description: C1-GFP time-lapse in T shape cell upon Nocodazole treatment Description: Centrin1-GFP live imaging in HeLa cell on a T micropattern upon inhibition of acto-myosin contractility via ML7. Time is in hrs:min. Frame rate is 30sec. Total acquisition length is 11min.

File Name: Supplementary Movie 11
Description: FUCCI HeLa time-lapse in Square shape cell Description: FUCCI HeLa cell live imaging on a Square micropattern. Red signal is hCdt1, a G1-to-S phase reporter and green is Gemin, a S-to-G2 phase marker. Time is in hrs:min. Frame rate is 5min. Total acquisition length is 20h11min.

File Name: Supplementary Movie 12
Description: FUCCI HeLa time-lapse in H shape cell. FUCCI HeLa cell live imaging on a H micropattern. Red signal is hCdt1, a G1-to-S phase reporter and green is Gemin, a S-to-G2 phase marker. Time is in hrs:min. Frame rate is 5min. Total acquisition length is 20h25min.

File Name: Supplementary Movie 13
Description: FUCCI HeLa time-lapse in Tripod shape cell. FUCCI HeLa cell live imaging on a Tripod micropattern. Red signal is hCdt1, a G1-to-S phase reporter and green is Gemin, a S-to-G2 phase marker. Time is in hrs:min. Frame rate is 10min. Total acquisition length is 21h30min.

File Name: Supplementary Movie 14
Description: FUCCI HeLa time-lapse in T shape cell. HeLa cell live imaging on a T micropattern. Red signal is hCdt1, a G1-to-S phase reporter and green is Gemin, a S-to-G2 phase marker. Time is in hrs:min. Frame rate is 2min. Total acquisition length is 17h38min.

File Name: Supplementary Movie 15
Description: FUCCI HeLa time-lapse in Square shape cell with 10µm blebbistatin. FUCCI HeLa cell live imaging on a Square micropattern upon 10µm blebbistatin treatment. Red signal is hCdt1, a G1-to-S phase reporter and green is Gemin, a S-to-G2 phase marker. Time is in hrs:min. Frame rate is 3min. Total acquisition length is 22h9min.

File Name: Supplementary Movie 16
Description: FUCCI HeLa time-lapse in H shape cell treated with 10µm blebbistatin. FUCCI HeLa cell live imaging on a H micropattern upon 10µm blebbistatin treatment. Red signal is hCdt1, a G1-to-S phase reporter and green is Gemin, a S-to-G2 phase marker. Time is in hrs:min. Frame rate is 3min. Total acquisition length is 18h.

File Name: Supplementary Movie 17
Description: FUCCI HeLa time-lapse in Tripod shape cell with 10µm blebbistatin .FUCCI HeLa cell live imaging on a Tripod micropattern upon 10µm blebbistatin treatment. Red signal is hCdt1, a G1-to-S phase reporter and green is Gemin, a S-to-G2 phase marker. Time is in hrs:min. Frame rate is 3min. Total acquisition length is 18h.

File Name: Supplementary Movie 18
Description: FUCCI HeLa time-lapse in T shape cell with 10µm blebbistatin. FUCCI HeLa cell live imaging on a T micropattern upon 10µm blebbistatin treatment. Red signal is hCdt1, a G1-to-S phase

reporter and green is Gemin, a S-to-G2 phase marker. Time is in hrs:min. Frame rate is 3min. Total acquisition length is 18h.

File Name: Supplementary Movie 19
Description: H2B-GFP HeLa time-lapse in Square shape cell. H2B-GFP and Brightfield live imaging of HeLa cells on a Square micropattern. Time is in hrs:min. Frame rate is 5min. Total acquisition length is 6h30min.

File Name: Supplementary Movie 20
Description: H2B-GFP HeLa time-lapse in H shape cell. H2B-GFP and Brightfield live imaging of HeLa cells on a H micropattern. Time is in hrs:min. Frame rate is 5min. Total acquisition length is 11h50min.

File Name: Supplementary Movie 21
Description: H2B-GFP HeLa time-lapse in Tripod shape cell. H2B-GFP and Brightfield live imaging of HeLa cells on a Tripod micropattern. Time is in hrs:min. Frame rate is 5min. Total acquisition length is 7h25min.

File Name: Supplementary Movie 22
Description: H2B-GFP HeLa time-lapse in T shape cell. H2B-GFP and Brightfield live imaging of HeLa cells on a T micropattern. Time is in hrs:min. Frame rate is 5min. Total acquisition length is 11h50min.
